# Renunciation of Homœopathy

**Published:** 1861-09

**Authors:** J. C. Peters


### Selections.

### RENUNCIATION OF HOMŒOPATHY.

By the late Editor of the North American Journal of Homœopathy.

*To the Editor of the American Medical Times.*

Sir :—I wish to put on record in your pages, not only that I have long since resigned all connection with any and every sectarian medical society and publication, but that I now most distinctly do not believe or practise according to any one medical dogma or exclusive system. I have repeatedly been on the point of making this declaration public in some regular medical journal, as it is well known that I have often done in private conversation and in homœopathic periodicals; but frequently the pressing demands of the sick have not left me time, and at other times I have been deterred by the urgent entreaties of friends, backed by that natural repugnance which every one has to publicly acknowledge a change of opinion.

In simple justice to myself, I will beg your indulgence to a short statement of my connection with homœopathy. When a mere school-boy, between twelve and fourteen years of age, and now I am forty-one, I was personally under the care of an aged and accomplished physician, Dr. Freytag, of Bethlehem, Penn. On my return from boarding-school to this my native city, I found many of my nearest relatives under the treatment of Drs. Gram and Gray. Thus, both in Pennsylvania and here, I was early thrown in contact with many and earnest converts to homœopathy. A short time spent in a wholesale drug store, opened my eyes to the immense amount of adulterated, spoiled, and poor drugs and medicines which were then and perhaps are now sold. Not a few of my dear est relatives had not been saved from agonizing death, and some were still suffering with varieties of the most distressing forms of chronic disease, which had not been averted by all the devotion and skill of many of the most accomplished physicians of the dominant school. I commenced the study of medicine, under the impression, and with the fervent hope that homœopathy, in its future and rational development, would supply all that was deficient in medicine; but all my natural instincts ever have been, and ever will be opposed to all bigoted exclusiveness and one-ideaism in religion, politics, science, and my much-loved profession. As far as lay in my power, I have never been unmindful for a day, from the commencement of my career as a medical student and practitioner, of the numerous and brilliant advances in regular medicine which have been constantly progressing, both in this country and abroad. I must say that I never have been a convert to the use of infinitesimal doses; they have been so repugnant to every fraction of common sense which I possess, that I have always felt absolutely degraded, when making, what I conceived to be, necessary trials with them. I have always felt that I was doing something foolish or wrong when giving them; that I was dealing with quantities so minute and so powerless, that it would be trifling with the lives of my friends and patients to depend upon them in serious cases, and with their time and comfort in milder attacks. I knew full well that Hahnemann had performed all his first cures with tangible doses, and had cited numerous instances from reliable medical authorities, in which, apparently, homœopathic cures had been effected with not unreasonably small doses. I determined to commence where he commenced, and if beaten back to the use of infinitesimal doses, would reluctantly, but at the same time decidedly follow the results of my experience. I have never felt myself obliged to fall back upon infinitesimal doses; but, on the contrary, have been more and more successful in strict proportion as I gradually increased upon the very small quantities which I first used, and in proportion as I departed from a slavish adherence to one system of medicine. The reports of others, both physicians and laymen, frequently led me to make careful trials of infinitesimal doses in various cases, but never with satisfactory success; while many extraordinary instances of recovery from distress and sickness in which no medicine had been given, and numerous consultations to which I was called by homœopathic physicians, in which severe disease had gone on unchecked by these powerless agents, more and more convinced me that they were irrational and unsafe.

A careful study of the Homœopathic Materia Medica, early convinced me that it was visionary and unreliable. I labored long and zealously to do my share towards giving it a more practical and common-sense shape.

The dogma, *similia similibus curantur*, was long a stumbling block to me; it seemed so utterly opposed to reason, that it was often with difficulty that I could force myself to practice according to it. But, many years ago I hit upon an explanation which was, and is still, perfectly satisfactory to me. It is self-evident that, in order to cure any disease, a state *different* from that presented by the disease, must be brought about; hence, a curative drug must either primarily or secondarily exert an *alterative* action; that is, if we leave mere revulsive effects out of the argument for the present. Similarity is not identity, but a similar thing, although it resembles somewhat, or even strongly, also *differs* somewhat, and even greatly. Hence, a drug which acts similarly to the action of any given disease, also differs somewhat in its action, and ultimately may exert an *alterative* effect. Similarity is a hybrid consisting of a great or greater degree of resemblance, coupled with a less or lesser amount of difference; in fact, similarity may be defined as a *slight degree of difference*, quite as well as interpreting it as a great degree of resemblance. Hence, the homœopathic law *is only an apparent and fragmentary truth*, not a complete exhaustive law. It is a fragment of the great law, *differentia differentiis curantur*, seu *alterantia alterantiis curantur*, of which in its form the old law, *contraria contrariis curantur*, is another fragment. For opposite or antagonistic things are such as *differ* in the greatest degree; while similar things are merely such as differ in the least, or lesser degree, or in certain particulars; while in others, they may be *essentially different.* Identity excludes the idea of difference, while similarity may include only the idea of casual likeness. Upon these ideas or principles, I have long thought, studied, and practiced, and have gradually become more and more convinced that the homœopathic is only a partially, or even only an apparently true law; a mere fragment of the greater law of alternative antagonistic action which has been practiced upon for ages.

The immense advances which have been made in the regular school in pathological anatomy, diagnosis, microscopical and chemical investigation, in auscultation and percussion, in the use of the speculum and ophthalmoscope, and in the use of ether and chloroform, necessarily force every student of medicine to give the larger portion of his attention to the publications of the dominant school. I have long endeavored to force these tangible, practical, and essential advances upon the attention of the homœopathic school, and labored almost in vain to convince the fraternity that the healing art is so far from having attained a state of perfection, that no school has a right, wholly, to despise and reject the other, and that a wholly derogatory estimation of every other method than their own, is not a necessary consequence of their adherence to the latter. Hence, I must prefer the greater to the lesser truth, and however painfully and reluctantly, must endeavor to cast my lot with other friends, other theories, and other practice.

But the homœopathists have discovered some new remedies, and renewed the use of many forgotten old ones. If consistent with the object of your periodical, at some future time, I will furnish short articles on the use of ignatia, cocculus, pulsatilla, agaricus, hammemalis, cannabis, sativa, euphrasia, and other remedies, simply premising that it is not at all necessary to use them in infinitesimal doses, nor always according to the homœopathic law.

Yours, &c.,

J. C. Peters, M. D.

